# Emerging Non-Breast Implant-Associated Lymphomas: A Systematic Review

**DOI:** 10.3390/cancers16234085

**Published:** 2024-12-05

**Authors:** Arianna Di Napoli, Santo Fruscione, Sergio Mazzola, Rosalba Amodio, Giorgio Graziano, Rita Mannino, Maurizio Zarcone, Giorgio Bertolazzi, Nicole Bonaccorso, Martina Sciortino, Daniele Domenico De Bella, Alessandra Savatteri, Miriam Belluzzo, Chiara Alba Norrito, Rosario Sparacino, Paolo Contiero, Giovanna Tagliabue, Claudio Costantino, Walter Mazzucco

**Affiliations:** 1 Pathology Unit, Department of Clinical and Molecular Medicine, Sant’Andrea University Hospital, Sapienza University of Rome, 00189 Rome, Italy; arianna.dinapoli@uniroma1.it; 2 PROMISE Department, University of Palermo, 90127 Palermo, Italy; nicole.bonaccorso@unipa.it (N.B.); martina.sciortino@unipa.it (M.S.); danieledomenico.debella@unipa.it (D.D.D.B.); alessandra.savatteri@unipa.it (A.S.); miriam.belluzzo@unipa.it (M.B.); chiaraalba.norrito@unipa.it (C.A.N.); rosario.sparacino@unipa.it (R.S.); claudio.costantino01@unipa.it (C.C.); walter.mazzucco@unipa.it (W.M.); 3 U.O.C. Epidemiologia Clinica con Registro Tumori, Azienda Ospedaliera Universitaria Policlinico di Palermo, 90127 Palermo, Italy; sergio.mazzola@policlinico.pa.it (S.M.); rosalba.amodio@policlinico.pa.it (R.A.); giorgio.graziano@policlinico.pa.it (G.G.); rita.mannino@policlinico.pa.it (R.M.); maurizio.zarcone@policlinico.pa.it (M.Z.); 4 Department of Economics, Business and Statistics, University of Palermo, 90127 Palermo, Italy; giorgio.bertolazzi@unipa.it; 5 PhD National Programme in One Health Approaches to Infectious Diseases and Life Science Research, Department of Public Health, Experimental and Forensic Medicine, University of Pavia, 27100 Pavia, Italy; 6 INT-IRCCS National Cancer Institute Foundation, 20133 Milan, Italy; paolo.contiero@istitutotumori.mi.it (P.C.); giovanna.tagliabue@istitutotumori.mi.it (G.T.)

**Keywords:** emerging lymphomas, anaplastic large-cell lymphoma, fibrin-associated large B-cell lymphoma, implants, medical devices

## Abstract

**Background:** Medical devices used for functional or esthetic purposes improve health and quality of life; however, they are not risk-free. Anaplastic large-cell lymphoma (ALCL), associated with breast implants, is a well-known and recognized distinct lymphoma entity. More recently, additional lymphomas have been reported in relation to prosthesis other than breast implants, as these allow the pericyte to develop into a clone that undergoes a maturation process, progressing toward full malignancy. **Methods:** We performed a systematic review with a descriptive analysis of data extracted from primary studies following PRISMA guidelines, including the search string “(IMPLANT* OR PROSTHES*) AND LYMPHOM*” in the PubMed, Scopus, Embase, and Google-Scholar databases. Data such as patient sex, age, implant site, prosthesis material, and lymphoma type were analyzed. Statistical methods, including Student’s *t*-test and Fisher’s exact test, were employed to compare lymphoma characteristics, with significance set at a *p*-value < 0.05. **Results:** From a total of 5992 studies, we obtained 43 case reports and series on a total of 52 patients diagnosed with prosthesis-associated lymphomas. The majority of implant-related lymphoma cases (85%) were of the B-cell type, mostly fibrin-associated large B-cell lymphoma (LBCL). This lymphoma type was more associated with biological (non-human-derived biological tissue), metallic, and synthetic implants (synthesized from non-organic components) (*p*-value = 0.007). Patients with ALCL had equal frequencies of metal and silicone prostheses (37.5%, 3 cases each), followed by synthetic prostheses (25%, 2 cases). ALCL cases were most common at skeletal (50%) and muscular-cutaneous sites (25%), whereas B-cell lymphomas were predominantly found in cardiovascular implants (50%), followed by skeletal (27%) and muscular-cutaneous (21%) sites. Death attributed to lymphoma took place in 67% of the cases, mostly LBCL occurring in cardiovascular sites. **Conclusions:** Because the included studies were limited to case reports and series, a potential non-causal link might have been documented between different implant materials, implant sites and lymphoma types. This underscores the importance of further comprehensive research and monitoring of non-breast implants.

## Simple Summary

Prostheses are highly beneficial as they reduce the need for formal healthcare and support services, but they are not without side effects. Despite advancements, issues such as implant rejection and perioperative complications persist, and new concerns have arisen, including the potential link between long-term prosthetic use and cancer, specifically lymphoma. While the overall risk of developing lymphoma from prosthetic implants remains low, it is essential to explore the factors contributing to this risk. This highlights the need for ongoing research into the safety and long-term effects of prosthetic devices. Based on this evidence, we conducted a descriptive analysis using individual patient data from primary studies to investigate the relationship between lymphomas and different types of prosthetic devices, excluding breast implants.

## 1. Introduction

Prosthetic devices have contributed to the improvement of countless lives, enabling people with physical impairments or functional limitations to live healthy, productive, independent, dignified lives, and to participate in the labor market and social life [1]. Prostheses are mainly used for functional or esthetic purposes, with breast, knee, hip, and vascular implants being the most common [2]. The use of prostheses can reduce the need for long-term care and caregivers, therefore representing devices of unquestionable usefulness; however, they are not free from side effects, despite the progress that has been made to prevent them [3]. Beyond implant rejection and perioperative complications, new side effects have been observed after several years of using prosthetic devices, including the occurrence of cancerous processes [4]. It is well established that prosthetic implants induce a foreign body response (FBR) which is a form of chronic inflammation resulting from the inflammatory reaction to a persistent foreign stimulus, and that chronic inflammation has been linked to cancer [5,6,7]. Inflammatory cells are indeed a critical source of various tumor-promoting inflammatory mediators. In particular, reactive oxygen species (ROS) and reactive nitrogen intermediates (RNIs) favor DNA damage, genomic instability, and the dysregulation of the epigenetic machinery, whereas cytokines and growth factors promote survival and the proliferation of transformed cells and create an immunosuppressive environment that decreases immunosurveillance of malignancy [6].The enormous variety of prosthetic materials and implant sites may result in high variability in the type of neoplastic occurrence (e.g., squamous-cell carcinoma, lymphomas, and sarcomas) [4,8]. Of these, lymphomas represent diseases that arise from a clonal proliferation of lymphocytes, documenting biologic heterogeneity and differences with respect to epidemiology, molecular and pathologic characteristics, clinical presentation, and optimal management [9].

The link between breast implants and anaplastic large-cell lymphoma (BIA-ALCL) has received significant attention in recent years [10]. In 2011, the Food and Drug Administration (FDA) highlighted the potential connection between breast implants and BIA-ALCL, consequently leading to efforts to monitor the distribution of the disease in association with specific types of breast implants [11,12]. In addition to BIA-ALCL, other B-cell lymphomas have been described in association with breast implants [13]. More recently, an increasing number of lymphoma cases associated with prostheses other than breast implants (POBIs) have been reported as well [14].

While the link between textured breast implants and ALCL is relatively well established [10], the evidence for other types of implants and lymphomas is less conclusive. The same general principles, chronic inflammation, immune dysregulation, and potential for particle release, could potentially apply to other situations, but the specific risks and mechanisms would likely vary depending on the implant material, location, and surface properties.

In orthopedic surgery, prosthesis-induced carcinogenesis remains an enigma. However, many reports have documented metal allergy or hypersensitivity-type responses to orthopedic implant debris, which has been histologically reported as ALVAL (aseptic lymphocyte-dominated vasculitis-associated lesion) [15]. This is characterized by tissue necrosis, fibrin, perivascular lymphocytes, and the formation of tertiary lymphoid organs (TLOs) containing B and T cells, and plasma cells [16]. Since TLOs usually develop in tissues involved by autoimmune diseases (i.e., rheumatoid arthritis, Sjogren syndrome), it has been suggested that the ions and nano-particles released by the implant may have the potential to form complexes with native proteins to generate an immunogenic complex leading to the local breakdown of self-tolerance and the generation of auto-reactive B cells [16]. Along with chronic inflammation, several systemic and organ-specific autoimmune conditions have been associated with an increased risk of developing lymphoma [17]. Moreover, as suggested for other large B-cell lymphomas arising in the context of long-standing chronic inflammation, the enclosed environment (the joint space and the space between the implant and the bone) may favor the immune escape, by local secretion of immunosuppressing cytokines (e.g., IL-10) and the expansion, by stimulation via autocrine or paracrine IL-6R engagement, of Epstein–Barr virus-transformed B cells [18,19,20,21].

Of interest, the World Health Organization (WHO) estimates that around 0.5% of the global population, which translates to approximately 35–40 million people, require prosthetics and orthotics services [22]. Therefore, despite the overall risk of developing lymphoma as a result of prosthetic implants remaining low, it is crucial to understand the potential factors and implications involved, prompting further investigation and raising important questions about the safety and long-term implications of certain prosthetic solutions [14].

According to the aforementioned evidence, we performed a descriptive analysis using individual patients’ data, extrapolated from primary studies available from the literature, with the aim of critically analyzing the relationship between lymphomas and different types of prosthetic devices and implants, excluding lymphomas associated with breast implants.

## 2. Materials and Methods

We conducted a systematic review of the existing scientific literature on the occurrence of lymphoma in patients with prostheses, using the PubMed, Scopus, Google Scholar, and EMBASE databases, including all studies published until September 2024. To define the research strategy, the Population, Exposure, Control and Outcomes (PECO) method was employed [23]. The search for articles started in September 2024 and the following search string “(IMPLANT* OR PROSTHES*) AND LYMPHOM*” was used. A descriptive analysis of data extracted from primary studies was then performed. The guidelines outlined in the Preferred Reporting Items for Systematic Reviews and Meta-Analyses statement (PRISMA) were followed [24]. The study protocol was registered in the PROSPERO international register (CRD42023435071).

The inclusion criteria comprised studies on lymphomas primarily related to the implantation of a prosthesis, with the exception of studies on lymphomas associated with breast implants (i.e., BIA-ALCL and EBV+ large B-cell lymphomas). Lymphomas diagnosed prior to prosthesis implantation or secondary lymphomas involving the implant site were excluded. Two independent reviewers conducted the research and selection of the studies. When a potentially relevant study was identified, the full-text article was independently evaluated by both reviewers. Any disagreements were resolved through discussion and consensus, and a third reviewer was involved if necessary. At the end, studies reporting on an individual basis information on the characteristics of lymphoma cases in patients who have received an implant, other than breast, were included in the statistical analyses. Incomplete data were treated as not available (NA), therefore analyses were performed on the available data only. Data extracted from each study were entered into an electronic database.

Since this analysis was based on published studies, it did not require ethical approval. However, we ensured adherence to ethical guidelines and principles regarding data confidentiality and anonymity.

A descriptive analysis of cases of lymphoma extracted from the selected primary studies was conducted. The variables obtained from the primary studies were sex, age at diagnosis, the site of the implant, prosthesis material, implant replacement, and the number of implant substitutions, the duration of the implant, disease signs and symptoms, the duration of symptoms, the type of lymphoma, its molecular characterization, the reason for implantation, the type of treatment, and the clinical outcomes at follow-up.

The different implantation sites were further grouped, according to the topographic similarities, into cardiovascular, muscular-cutaneous (gluteus, thigh, eye, cheek, pacemaker pocket in chest), skeletal (osteoarticular), and other sites (e.g., stomach and eye).

For quantitative variables we calculated were the mean and median values, the standard deviations, and the ranges, while for categorical analysis, the variables’ absolute and relative frequencies were provided. We compared different groups based on the type of diagnosed lymphoma (large B-cell lymphoma (LBCL), extranodal marginal-zone lymphoma of mucosa-associated lymphoid tissue (EMZL), and anaplastic large-cell lymphoma), prosthesis material at the first implant (metal, silicone, synthetic synthesized from non-organic components, biological originating from non-human species), and the site of the implant (cardiovascular, muscular-cutaneous, skeletal, and other sites). Follow-up analyses were performed comparing the following groups of patients: deceased, with refractory/relapsed disease (R/R,), and in complete remission (CR), excluding patients who died due to causes other than lymphoma. To assess any statistical significance, we employed Student’s *t*-test for continuous variables, except for the onset time for which a non-parametric median test, that takes into account the asymmetry of its distribution, was more appropriate. Fisher’s exact test was applied for qualitative variables [25]. The significance level was set at a *p*-value of less than 0.05. For the statistical analysis, R software was used. We performed a quality assessment to evaluate the methodological quality of case reports and case series based on the domains of selection, ascertainment, causality, and reporting (Supplementary Table S2).

## 3. Results

**Figure 1:**
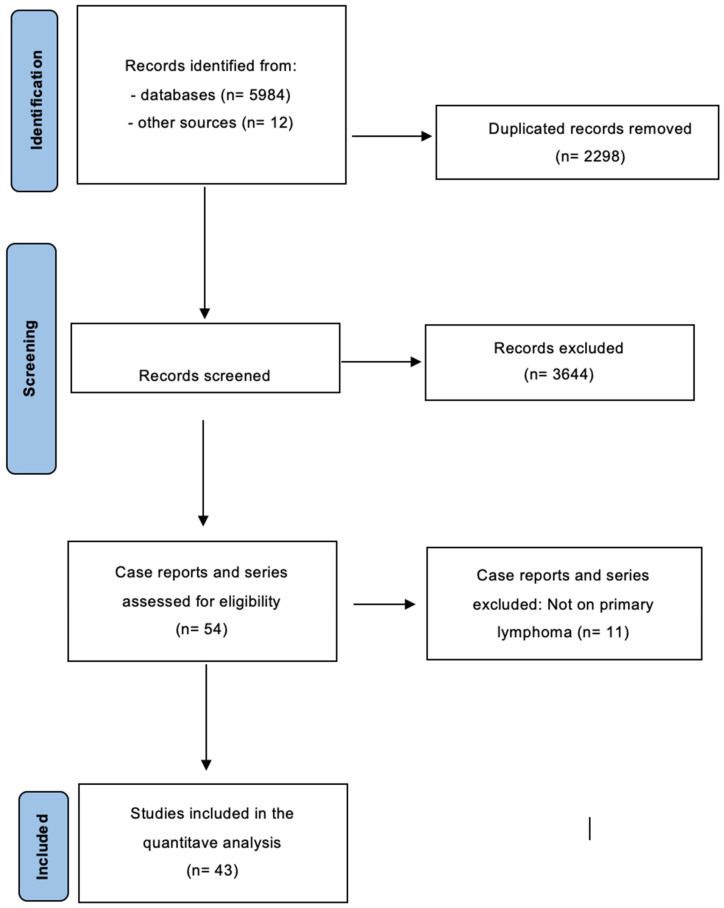
Flow diagram for the selection of the primary studies on lymphoma associated with prosthesis (other than BIA-ALCL) included in the study.

The initial search identified 5992 studies on prostheses and lymphomas (Figure 1).

Of the 5992 identified studies, 3644 mentioned lymphoma or prostheses separately and were then excluded. After eliminating duplicates and articles that did not match the inclusion criteria, 55 case reports and case series were selected and consulted. Another 11 studies were further excluded because they did not include primary lymphoma but secondary involvement by lymphomas originated from other sites. A total of 43 case reports and series articles were selected and 52 patients diagnosed with prosthesis-associated lymphomas were considered for the statistical analysis [26,27,28,29,30,31,32,33,34,35,36,37,38,39,40,41,42,43,44,45,46,47,48,49,50,51,52,53,54,55,56,57,58,59,60,61,62,63,64,65].

**Figure 2:**
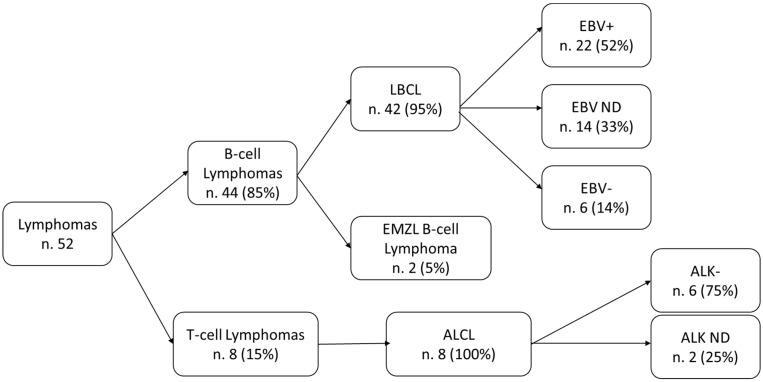
Description of the cases extracted from the selected primary studies, by cell lineage, lymphoma type, and molecular characterization (EBV for LBCL; ALK for ALCL). Legend: LBCL: large B-cell lymphoma; ALCL: anaplastic large-cell lymphoma; ND: not defined.

Figure 2 summarizes the description of the cases included in the study, according to lymphoma type.

Of the 52 cases in the study, males (34; 65%) had a mean age at diagnosis of 62 years old (median age: 65; range: 23–85), with a mean time to prosthesis-associated lymphoma onset from the date of implantation of 9 years (range: 0.2–32), while females (18; 35%) presented a mean age at diagnosis of 67 years old (median age: 72; range: 30–80) (*p*-value = 0.219), with a mean time to prosthesis-associated lymphoma onset from the date of the first implantation of 11 years (range: 1–50) (*p*-value = 0.440).

Forty-four out of the fifty-two (81%) cases were B-cell lymphomas, with 42 large B-cell lymphomas (LBCLs) and two extra-nodal marginal-zone lymphomas. Among the LBCL cases tested for Epstein–Barr virus (EBV) infection, 22 out of 28 (82%) were EBV-positive, while 6/28 (18%) were EBV-negative. According to the most recent lymphoma classification of the World Health Organization (WHO), 23 (55%) LBCL cases with lymphoma cells described in association with fibrin were considered as fibrin-associated large B-cell lymphomas (FA-LBCLs), whereas for the remaining 19 cases the presence of fibrin was not reported [9,26]. Eight cases (15%) were T-cell lymphomas and all of them were anaplastic large-cell lymphomas (ALCLs), in particular anaplastic lymphoma kinase (ALK)-negative cases, while for two cases the ALK status was not reported. One case, classified as “undefined cell lymphoma”, was excluded from the analyses.

Compared to patients with B-cell lymphomas, those with ALCLs had a slightly lower mean and median age at diagnosis (mean age: 61 vs. 64 years; median age: 63 vs. 68 years) and a shorter mean but similar median time from implantation to diagnosis (mean: 7 vs. 10 years; median: 6 vs. 7 years). However, no statistically significant differences were observed (*p*-value > 0.05). Males were more frequently represented in both ALCL and B-cell lymphoma cases (Table 1).

**Figure 3:**
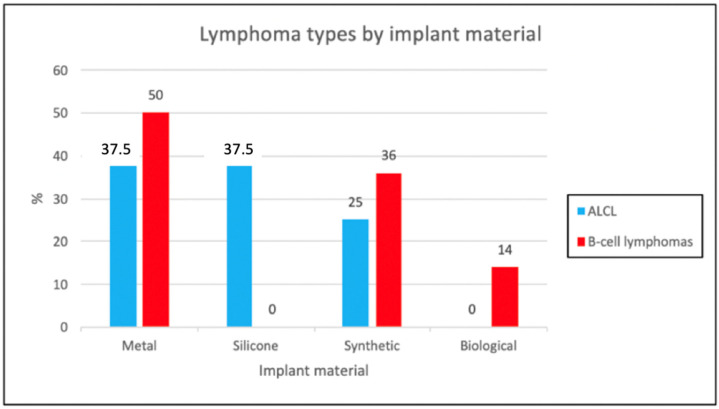
Bar chart showing lymphoma types by implant material.

Concerning the materials used in the prostheses, metal was the most commonly reported, with 25 cases (48%), followed by synthetic materials in 18 cases (35%), biological materials in 6 cases (11%), and silicone in 3 cases (6%) (Table 2) (Figure 3).

Specifically, among patients with ALCL, metal and silicone prostheses were reported at the same frequency (37.5%, three cases each), followed by synthetic prostheses (25%, two cases). For patients with B-cell lymphomas, metal prostheses were more frequently used (50%, 22 cases), followed by synthetic prostheses (36%, 16 cases), and biological materials (14%, 6 cases). Notably, biological prostheses were reported in B-cell lymphoma cases only, while silicone prostheses were exclusively associated with ALCL. These associations between prosthesis material and lymphoma types presented as statistically significant (*p*-value = 0.007) (Table 2).

**Figure 4:**
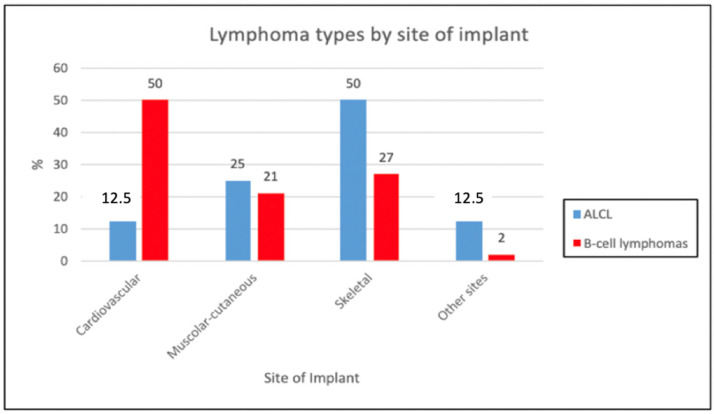
Bar chart showing lymphoma types by site of implant.

The distribution of the different sites of implant was explored for the two lymphoma types (Table 2). The most frequent site of implant was the cardiovascular system (23 cases; 44%), followed by the skeletal system (16 cases; 31%) and the muscular-cutaneous (11 cases; 21%), while the two (4%) remaining prostheses were implanted in the stomach and the eye, respectively (Figure 4).

Regarding the lymphoma type, the skeletal prostheses were the most prevalent in patients diagnosed with ALCL (50%, four cases), followed by muscular-cutaneous (25%, two cases), cardiovascular (12.5%, one case), and other sites (12.5%, one case). Conversely, among patients with B-cell lymphomas the cardiovascular prostheses were the most frequently reported (50%, 22 cases), followed by skeletal prostheses (27%, 12 cases), muscular-cutaneous (21%, 9 cases), and other sites (2%, 1 case); however, no statistically significant difference was highlighted (*p*-value = 0.09).

Follow-up data were available for 45 patients with an implant-associated lymphoma. Seven patients died for causes not related to the lymphoma, such as breast cancer, infections, or surgical complications. Of the remaining 38 cases (Table 3), 14 (37%) were reported to have died (4 cases) or with relapsed or resistant disease (R/R, 4 cases), while 20 (53%) patients were in complete remission (CR). At diagnosis, the deceased patients had a mean age of 60 years old (median 64; range: 23–85).

The deceased patients (14) had a diagnosis-to-death latency time of 12 months (range <0.2–36 months). Fourteen of the 20 patients in CR received chemotherapy and/or radiotherapy, and the mean time to being disease-free from diagnosis, calculated at the last available follow-up, was of 15 months (range: 1–36 months).

No significant difference (*p*-value = 0.66) was found for the clinical outcomes (CR versus deceased or R/R disease), based on prosthetic material (Table 3). Patients with synthetic and silicone prostheses were almost equally represented in both groups, whereas a slightly higher rate of patients in CR were found among the biological (67% in CR versus 33% deceased or R/R) or metal-implanted (60% in CR versus 35% deceased or R/R) patients. Similarly, when considering the site of implant, no significant association was found (*p*-value = 0.69), although cardiovascular prostheses were more frequently associated with deaths or R/R diseases (60%), whereas skeletal ones with CR (64%) (Table 3). Based on lymphoma type, three of the four (75%) ALCL patients were disease-free, whereas 50% of the B-cell lymphoma patients died or had a relapsed/resistant disease (*p*-value = 0.77). Among the LBCL cases, 57% (24/42 cases) were FA-LBCL and all the investigated cases were EBV+ (22/22 cases). FA-LBCL more frequently involved the cardiovascular system (18/24 cases, 75%) and the use of synthetic materials (14/24 cases, 58%). Twelve patients died (8/10, 67%) or had R/R diseases (4/10, 33%) and the majority of them were associated with cardiovascular involvement (9/12 cases, 75%). In particular, when considering the cardiovascular site only, three patients died of surgical complications, six died of lymphoma, three had R/R (relapsed/refractory) disease, and four were in CR.

## 4. Discussion

Prosthetic devices have immense benefits for patients, but they are not free from potential risks. While breast implant-associated lymphoma represents one of the most well-known cancers associated with medical implants, to date there is scant evidence linking the use of other prosthetic devices to lymphoma or related complications [12,14].

Therefore, we conducted a descriptive analysis using individual patients’ data, obtained from primary published studies on lymphoma cases occurring in patients receiving a medical prosthetic device or implant, trying to improve our knowledge on any potential relationship between the occurrence of lymphomas and the different types of prosthetic medical devices and implants, excluding breast implants.

In brief, in our series males with a prosthetic-associated lymphoma were younger than women. The distribution of lymphoma cases in patients receiving a prosthetic device or implant for medical treatments revealed that B-cell lymphomas were predominant, as compared to ALCLs. These data sit in contrast with what has been reported to date for breast implant-associated lymphomas, in which ALCL largely predominated [12,66]. Furthermore, ALCL patients had a shorter, but not significant, mean time from implantation to diagnosis than patients with B-cell lymphomas.

More than half of the B-cell lymphomas were FA-LBCL (57%), which consists of non-mass-forming aggregates of usually EBV+ neoplastic B cells in a background of fibrin, developing in confined natural or acquired spaces, with many cases reported in association with prosthetic devices [9,41,56,60,62,67]. In this setting, local chronic inflammation elicited by the implant is thought to favor immune evasion of EBV-transformed B cells. Similarly, the chronic antigenic stimulation given by the prosthetic material could favor the proliferation, and eventual transformation, of EBV-negative B cells by the accumulation of genomic alterations. Therefore, molecular studies are warranted to understand the mechanisms behind the pathogenesis of non-breast implant-associated lymphomas.

A significantly different distribution of lymphoma cases according to both prosthesis material and lymphoma type was found. In particular, ALCL cases were more frequently reported in patients implanted with metal, silicone, and synthetic prostheses, with no biological material cases, while metal, synthetic and biological implants predominated in B-cell lymphoma patients (***p***-value: 0.007), with no cases associated with silicone. However, it has to be emphasized that B-cell lymphomas associated with breast implants were excluded from our research. Indeed, when adding the 15 B-cell lymphomas reported to date in the literature in patients with breast implants, the difference with ALCL would lose its significance [68]. Coherently, the cardiovascular and skeletal site of the implant more frequently involved in the B-cell lymphoma cases, likely due to the frequent use of synthetic/biological materials in cardiovascular surgery and of metal prosthesis for orthopedic and pacemaker implantations [44]. In addition, the occurrence of lymphomas following the implantation of cardiac and skeletal prostheses seriously challenges the balance between cost and benefit. These types of prostheses are essential for the survival of the individual, making their replacement necessary but also risky due to the patient’s fragile health condition.

From our analysis the follow-up data available showed that 67% of the patients experienced death attributed to lymphoma (14/21 of all deaths), indicating the severity and impact of the disease. All but one was LBCL, yet no significant association between the clinical outcome and the prosthetic material or anatomical involved site was observed.

However, when considering the FA-LBCL cases only, 12 patients died or had R/R disease, and the majority of them were associated with cardiovascular involvement (75%). In the literature, several FA-LBCL cases have been reported with the presence of prosthetic devices and the prognosis seems to be associated with the involved site [41,56,60,62]. Our data support the hypothesis that FA-LBCL arising in association with cardiovascular implants might be linked to more severe complications and to higher mortality rates.

It is important to acknowledge the limitations of this study, such as the relatively small sample size, the availability of case reports and series only, and the potential confounding factors that could have influenced our findings. The small sample size, in particular regarding ALCL cases, may have affected our analyses, especially when comparing the different types of lymphomas. Both selection and reporting bias of the different primary studies included in the analyses cannot be excluded; therefore, potentially introducing an unmeasurable heterogeneity of data across the included case reports and series. Lastly, due to incompleteness or unavailability of information, therapies, follow-up, and comorbidities were not taken into account in the analyses. Greater uniformity in the studies would allow for more reliable comparisons in larger future studies that should focus on these aspects. Future research should focus on collecting standardized data on comorbidities, treatments, and follow-up to ensure consistency and reliability.

## 5. Conclusions

In conclusion, this study did not allow us to establish any causal relationship between the different implant materials or sites of implantation and the different lymphoma cell types, but rather highlighted some potential connections, suggesting the need of further investigations in the epidemiological and molecular fields with larger sample sizes, controlled designs, and comprehensive data collection. Recognizing the link between lymphoma and implantation also carries therapeutic implications. Alongside the traditional pharmacological approach, surgical removal of the prosthesis—and potential replacement if essential to the patient’s survival—should be considered in these cases. Moving forward, it will be important to carefully select the materials used in prosthesis construction, while in the present, the close monitoring of individuals with prostheses is necessary.

In order to elucidate the current knowledge in this field and to fill the existing knowledge gaps, while establishing a clearer understanding of the potential risks, future research efforts should be addressed also at the monitoring of the epidemiology of implant-associated lymphomas through the strengthening of interoperability between cancer registers and prosthetic registers [69]. Improving interoperability between cancer registries and prosthesis registries would increase the sample by capturing more cases of prosthesis-related lymphomas, facilitating larger studies that could yield stronger evidence.

## Figures and Tables

**Table 1:** The characteristics of the 52 patients receiving a medical prosthetic device or implant (other than a breast implant), by lymphoma type.

	Anaplastic Large-Cell Lymphoma	B-Cell Lymphomas	*p*-Value
n. (%)	8 (15%)	44 (85%)	-
M	5	29	0.99
F	3	15	
Mean age at diagnosis	61	64	0.50
Median age at diagnosis	63	68	0.25
Male–female	5:3	29:15	-
Mean time to diagnosis from implantation	7	10	0.17
Median time to diagnosis from implantation	6	7	0.44

**Table 2:** The distribution of the 52 lymphomas associated with non-breast implants according to lymphoma type, by prosthetic material (a) and site (b).

(a) Prosthesis Material	ALCL (%)	B-Cell Lymphomas (%)	Total (%)	*p*-Value
Metal	3 (37.5)	22 (50)	25 (48)	0.007
Cardiovascular	-	2 (9)	2 (8)	
Muscular-cutaneous	-	7 (6 pacemaker pockets; 1 thigh) (32)	7 (27)	
Skeletal	3 (100%)	12 (55)	15 (61)	
Other sites	-	1 (eye) (5)	1 (4)	
Silicone	3 (37.5)	0 (0)	3 (6)	
Cardiovascular	-	-		
Muscular-cutaneous	2 (gluteal) (67)	-	2 (67)	
Skeletal	-	-	-	
Other sites	1 (stomach) (33)	-	1 (33)	
Synthetic	2 (25)	16 (36)	18 (35)	
Cardiovascular	1 (50)	15 (94)	16 (89)	
Muscular-cutaneous	-	1 (chest) (6)	1 (5.5)	
Skeletal	1 (50)	-	1 (5.5)	
Other sites	-	-	-	
Biological	0 (0%)	6 (14)	6 (11)	
Cardiovascular	-	5 (83)	5 (83)	
Muscular-cutaneous	-	1 (cheek) (17)	1 (17)	
Skeletal	-	-	-	
Other sites	-	-	-	
Total	8 (100)	44 (100)	52 (100)	
**(b) Prosthesis Site of Implant**	**ALCL (%)**	**B-Cell Lymphomas (%)**	**Total (%)**	***p*-Value**
Cardiovascular	1 (12.5)	22 (50)	23 (44)	0.09
Muscular-cutaneous	2 (25)	9 (21)	11 (21)	
Skeletal	4 (50)	12 (27)	16 (31)	
Other sites	1 (12.5)	1 (2)	2 (4)	
Total	8 (100)	44 (100)	52 (100)	

Fisher’s exact test applied on the values in the gray rows.

**Table 3:** Follow-up data on 38 patients with an implant-associated lymphoma.

	Death (%)	Relapsed/Refractory Disease (R/R) (%)	Complete Remission (CR) (%)	Total (%)	*p*-Value
Lymphoma type					
ALCL	1 (25)	0 (0)	3 (75)	4 (100)	0.77 *
B-cell lymphomas	13 (38)	4 (12)	17 (50)	34 (100)	
Total	14 (37)	4 (10)	20 (53)	38 (100)	
Type of material					
Metal	7 (35)	1 (5)	12 (60)	20 (100)	0.66 *
Silicone	1 (50)	-	1 (50)	2 (100)	
Synthetic	4 (31)	3 (23)	6 (46)	13 (100)	
Biological	2 (67)	-	1 (33)	3 (100)	
	14 (37)	4 (10)	20 (53)	38 (100)	
Total					
Site of implant					
Cardiovascular	6 (40)	3 (20)	6 (40)	15 (100)	0.69 *
Muscular-cutaneous	4 (40)	1 (10)	5 (50)	10 (100)	
Skeletal	4 (36)	-	7 (64)	11 (100)	
Other sites	-	-	2 (100)	2 (100)	
Total	14 (37)	4 (10)	20 (53)	38 (100)	
Patient age (years)					
Mean	60	66	62		0.81 *
Median	64	68	66		
Range	23–85	56–72	25–81		

* Calculated between death or R/R patients versus patients in CR (complete remission).

## Data Availability

The data presented in this study are available on request from the corresponding author.

## Supplementary Materials

The following supporting information can be downloaded at: https://www.mdpi.com/article/10.3390/cancers16234085/s1, Table S1. Summary table containing all cases extracted from primary studies (case reports, case series) that have been included in the analyses; Table S2. Quality assessment of all articles that have been included in the systematic review.
