# Supplementary material for: Emerging Non-Breast Implant-Associated Lymphomas: A Systematic Review
Source: Cancers (Basel). 2024 Dec 5;16(23):4085. doi: 10.3390/cancers16234085 (PMC11640267; doi:10.3390/cancers16234085)
Supplement: Supplementary file 1 [file cancers-16-04085-s001.zip › cancers-3291457-supplementary.pdf]

Supplementary Table S1. Summary table containing all cases extracted from primary studies (case reports, case series) that have been included in the analyses.

Authors	Title	n. of Participants	Sex	Age	Type of Prosthesis	Specific Prosthesis Material	Type of Lymphoma
Parkhi M [26]	Non-mammary implant associated anaplastic large cell lymphoma.	1	M	63	Metal	Metal	ALCL
Wang SJ [27]	Development of Primary Diffuse Large B-Cell Lymphoma Around an Internal Fixation Implant After Lumbar Fusion Surgery: A Case Report and Review of the Literature.	1	M	63	Metal	Metal	DLBCL
Palraj B [28]	Soft tissue anaplastic large T-cell lymphoma associated with a metallic orthopedic implant: case report and review of the current literature	1	M	77	Metal	Metal	ALCL
Tuck M [29]	Anaplastic large cell lymphoma masquerading as osteomyelitis of the shoulder: an uncommon presentation	1	M	50	Synthetic, Suture Joint	Suture Joint	ALCL
Cheuk W [30]	Metallic implant-associated lymphoma: a distinct subgroup of large B-cell lymphoma related to pyothorax-associated lymphoma?.	1	M	56	Metal	Metal: cobalt-chromium-molybdenum alloy	DLBCL
McDonald I [31]	Malignant lymphoma associated with internal fixation of a fractured tibia.	1	M	48	Metal	Metal	ALCL
Dodion P [32]	Immunoblastic lymphoma at the site of an infected vitallium bone plate	1	M	49	Metal	Stainless titanium	DLBCL
Mendes J Jr [33]	Gluteal Implant-Associated Anaplastic Large Cell Lymphoma	1	F	63	Silicone	silicone implant	ALCL
Shauly O [34]	The First Reported Case of Gluteal Implant-Associated Anaplastic Large Cell Lymphoma (ALCL).	1	F	49	Silicone	bilateral textured silicone gluteal implants	ALCL
Menter T [35]	ALK-negative anaplastic large cell lymphoma arising in the thrombus of an aortic prosthesis preceeded by clonally related lymphomatoid papulosis	1	M	73	Synthetic	EVAR Dacron	ALCL
Manikkam Umakanthan J [36]	Bariatric Implant-Associated Anaplastic Large-Cell Lymphoma	1	F	63	Silicone	Silicone-based elastopolymers	ALCL
Rajeev A [37]	Diffuse B Cell Non-Hodgkin's Lymphoma Presenting Atypically as Periprosthetic Joint Infection in a Total Hip Replacement	1	F	77	Metal	Metal	DLBCL
Sunitsch S [38]	Case Report: Epstein-Barr-Virus negative diffuse large B-cell lymphoma detected in a peri-prosthetic membrane	1	F	80	Metal	Metal	DLBCL
Farah FJ [39]	Recurrent primary cardiac lymphoma on aortic valve allograft: implications for therapy	1	M	60	Biological	Allograft	DLBCL
Albat B [40]	Surgical treatment for primary lymphoma of the heart simulating prosthetic mitral valve thrombosis	1	F	66	Synthetic	St. Jude mitral valve	DLBCL
Miller DV [41]	Epstein-Barr virus-associated diffuse large B-cell lymphoma arising on cardiac prostheses	3	M	48	Metal	Bjork-Shiley mechanical valve	DLBCL
Miller DV [41]	Epstein-Barr virus-associated diffuse large B-cell lymphoma arising on cardiac prostheses	3	F	80	Biological	Biological (bovine pericardial)	DLBCL
Miller DV [41]	Epstein-Barr virus-associated diffuse large B-cell lymphoma arising on cardiac prostheses	3	F	79	Synthetic	synthetic tube graft	DLBCL
Berrio G [42]	Diffuse Large B-Cell Lymphoma Associated with a Chronic Inflammatory Condition Induced by Metallic Implants: A Case Report	1	M	60	Biological	Allograft	DLBCL

Sanchez-Gonzalez B [43]	Diffuse large B-cell lymphoma associated with chronic inflammation in metallic implant	1	M	66	Metal	Metal	DLBCL
Chaudhry MS [44]	Diffuse large B cell lymphoma complicating total knee arthroplasty: case report and literature review of the association of diffuse large B cell lymphoma with joint replacement	1	M	76	Metal	Metal	DLBCL
Fujimoto M [45]	EBV-associated diffuse large B-cell lymphoma arising in the chest wall with surgical mesh implant	1	M	61	Synthetic	PET surgical mesh	DLBCL
O'Shea K [46]	Periprosthetic malignancy as a mode of failure in total hip arthroplasty	1	F	75	Metal	Metal	DLBCL
Dürrleman NM [47]	Cardiac lymphoma following mitral valve replacement	1	F	65	Synthetic	Dacron	DLBCL
Syed AA [48]	Osseous malignant non-Hodgkin's B-cell lymphoma associated with total hip replacement	1	F	75	Metal, Synthetic	stainless steel; polyethylene; cement	DLBCL
Radhi JM [49]	Soft tissue malignant lymphoma at sites of previous surgery.	3	M	25	Metal	Metal	DLBCL
Radhi JM [49]	Soft tissue malignant lymphoma at sites of previous surgery.	3	F	63	Metal	Metal	DLBCL
Radhi JM [49]	Soft tissue malignant lymphoma at sites of previous surgery.	3	F	45	Metal	Metal	DLBCL
Di Nisio LA [50]	Eyelid lymphoma in a patient with a gold weight implant. Linfoma palpebral en paciente con implante de pesa de oro.	1	M	72	Metal	Gold	Indolent B cell Lymphoma
Yohei Yamamoto [51]	Fibrin-associated diffuse large B-cell lymphoma arising in an abdominal aortic graft: A case report and literature review,	1	M	72	Synthetic	Dacron	DLBCL
Lee SH [52]	B-Cell Lymphoma in a Patient With a History of Foreign Body Injection	1	F	72	Biological	Dermal Filler	Indolent B cell Lymphoma
Ito H [53]	B Malignant lymphoma at the site of a total hip replacement	1	F	80	Metal, Synthetic	Iron-chromium-nichel-polyethylene	DLBCL
Afrapoli AH [54]	Heart lymphoma 6 years after a prosthetic ascending aorta replacement: related or unrelated?.	1	F	ND	Synthetic	Dacron	DLBCL
Hamour SM [55]	B-cell lymphoma arising around a PTFE graft.	1	F	77	Synthetic	Polytetrafluoroethylene	DLBCL
Hojo N [56]	Non-Hodgkin's lymphoma developing in a pacemaker pocket.	1	M	29	Metal	Pacemaker	DLBCL
Moruzzo D [57]	A rare case of non-Hodgkin lymphoma in a pacemaker pocket	1	M	68	Metal	Pacemaker	DLBCL
Nemec J [58]	B-Cell lymphoproliferative disorder of an ICD pocket: a diagnostic puzzle in an immunosuppressed patient.	1	M	70	Metal	Pacemaker	DLBCL
Patris V [59]	Primary B-cell lymphoma developing at epicardial pacemaker lead site [published correction appears in *J Card Surg*. **2015**;30(5):476	1	M	85	Metal	Pacemaker	DLBCL
Keyser A [60]	B-Cell lymphoma at the site of pacemaker generator	1	M	81	Metal	Pacemaker	DLBCL
Gruver AM [61]	Fibrin-associated large B-cell lymphoma: part of the spectrum of cardiac lymphomas	1	M	55	Synthetic	synthetic tube graft	DLBCL
Boyer DF [62]	Fibrin-associated EBV-positive Large B-Cell Lymphoma: An Indolent Neoplasm With Features Distinct From Diffuse Large B-Cell Lymphoma Associated With Chronic Inflammation	3	M	56	Synthetic	synthetic tube graft	DLBCL
Boyer DF [62]	Fibrin-associated EBV-positive Large B-Cell Lymphoma: An Indolent Neoplasm With Features Distinct From Diffuse Large B-Cell	3	M	68	Synthetic	synthetic tube graft	DLBCL

	Lymphoma Associated With Chronic Inflammation						
Boyer DF [62]	Fibrin-associated EBV-positive Large B-Cell Lymphoma: An Indolent Neoplasm With Features Distinct From Diffuse Large B-Cell Lymphoma Associated With Chronic Inflammation	3	M	71	Synthetic	synthetic tube graft	DLBCL
Di Napoli A [63]	Cavity-based lymphomas: challenges and novel concepts. A report of the 2022	6	F	30	Synthetic	Goretex	DLBCL
Di Napoli A [63]	Cavity-based lymphomas: challenges and novel concepts. A report of the 2022	6	M	23	Biological, Metal	Melody valve	DLBCL
Di Napoli A [63]	Cavity-based lymphomas: challenges and novel concepts. A report of the 2022	6	M	67	Metal	Metal	DLBCL
Di Napoli A [63]	Cavity-based lymphomas: challenges and novel concepts. A report of the 2022	6	M	72	Synthetic	Synthetic	DLBCL
Di Napoli A [63]	Cavity-based lymphomas: challenges and novel concepts. A report of the 2022	6	M	64	Synthetic, Metal	Synthetic, Metal	DLBCL
Di Napoli A [63]	Cavity-based lymphomas: challenges and novel concepts. A report of the 2022	6	M	72	Metal	Pacemaker	DLBCL
Raupach J [64]	Endovascular Treatment of Symptomatic Thoracic Aneurysm Due to Periaortic Lymphoma.	1	M	74	Synthetic	Nitinol and sintetic material	DLBCL
Bell D [65]	Diffuse Large B Cell Lymphoma in a Prosthetic Aortic Graft.	1	M	70	Synthetic	Hall valve and Polyethylene terephthalate (Dacron) graft	DLBCL

Supplementary Table S2. Quality assessment of all articles that have been included in the systematic review.

Authors	Sex	Age	Implant Site	Prosthesis Material	Years of Implant	Lymphoma Type	Treatment	Follow-Up	Score
Di Napoli A [63]	Yes	Yes	Yes	Yes	No	Yes	No	No	5
Parkhi M [26]	Yes	Yes	Yes	Yes	Yes	Yes	No	No	6
Wang SJ [27]	Yes	Yes	Yes	Yes	Yes	Yes	No	No	6
Palraj B [28]	Yes	Yes	Yes	Yes	Yes	Yes	No	No	6
Dodion P [32]	Yes	Yes	Yes	Yes	Yes	Yes	No	No	6
Mendes J Jr [33]	Yes	Yes	Yes	Yes	Yes	Yes	No	No	6
Shauly O [34]	Yes	Yes	Yes	Yes	Yes	Yes	No	No	6
Menter T [35]	Yes	Yes	Yes	Yes	Yes	Yes	No	No	6
Rajeev A [37]	Yes	Yes	Yes	Yes	Yes	Yes	No	No	6
Farah FJ [39]	Yes	Yes	Yes	Yes	Yes	Yes	No	No	6
Miller DV [41]	Yes	Yes	Yes	Yes	Yes	Yes	No	No	6
Berrio G [42]	Yes	Yes	Yes	Yes	No	Yes	No	No	6
Fujimoto M [45]	Yes	Yes	Yes	Yes	Yes	Yes	No	No	6
O'Shea K [46]	Yes	Yes	Yes	Yes	Yes	Yes	No	No	6
Dürrleman NM [47]	Yes	Yes	Yes	Yes	Yes	Yes	No	No	6
Radhi JM [49]	Yes	Yes	Yes	Yes	Yes	Yes	No	No	6
Ito H [53]	Yes	Yes	Yes	Yes	Yes	Yes	No	No	6
Afrapoli AH [54]	Yes	No	Yes	Yes	Yes	Yes	No	No	6
Moruzzo D [57]	Yes	Yes	Yes	Yes	Yes	Yes	No	No	6
Patris V [59]	Yes	Yes	Yes	Yes	Yes	Yes	No	No	6
Boyer DF [62]	Yes	Yes	Yes	Yes	Yes	Yes	No	No	6
Raupach J [64]	Yes	Yes	Yes	Yes	Yes	Yes	No	No	6
Sanchez-Gonzalez B [43]	Yes	Yes	Yes	Yes	Yes	Yes	Yes	No	7
Chaudhry MS [44]	Yes	Yes	Yes	Yes	Yes	Yes	Yes	No	7
Yohei Yamamoto [51]	Yes	Yes	Yes	Yes	Yes	Yes	No	No	7
Keyser A [60]	Yes	Yes	Yes	Yes	Yes	Yes	No	Yes	7
Gruver AM [61]	Yes	Yes	Yes	Yes	Yes	Yes	No	Yes	7
Tuck M [29]	Yes	Yes	Yes	Yes	Yes	Yes	Yes	Yes	8
Cheuk W [30]	Yes	Yes	Yes	Yes	Yes	Yes	Yes	Yes	8
McDonald I [31]	Yes	Yes	Yes	Yes	Yes	Yes	Yes	Yes	8
Manikkam Umakanthan J [36]	Yes	Yes	Yes	Yes	Yes	Yes	Yes	Yes	8
Sunitsch S [38]	Yes	Yes	Yes	Yes	Yes	Yes	Yes	Yes	8
Albat B [40]	Yes	Yes	Yes	Yes	Yes	Yes	Yes	Yes	8
Syed AA [48]	Yes	Yes	Yes	Yes	Yes	Yes	Yes	Yes	8
Di Nisio LA [50]	Yes	Yes	Yes	Yes	Yes	Yes	Yes	Yes	8
Lee SH [52]	Yes	Yes	Yes	Yes	Yes	Yes	Yes	Yes	8
Hamour SM [55]	Yes	Yes	Yes	Yes	Yes	Yes	Yes	Yes	8
Hojo N [56]	Yes	Yes	Yes	Yes	Yes	Yes	Yes	Yes	8
Nemec J [58]	Yes	Yes	Yes	Yes	Yes	Yes	Yes	Yes	8
Bell D [65]	Yes	Yes	Yes	Yes	Yes	Yes	Yes	Yes	8
